# Neutrophil Extracellular Traps in Cardiovascular Diseases: Pathological Roles and Therapeutic Implications

**DOI:** 10.3390/biom15091263

**Published:** 2025-09-01

**Authors:** Yan Ma, Jun Zhang, Yaxuan Qi, Yating Lu, Yalan Dong, Desheng Hu

**Affiliations:** 1 Department of Integrated Traditional Chinese and Western Medicine, Union Hospital, Tongji Medical College, Huazhong University of Science and Technology, Wuhan 430022, China; m202376304@hust.edu.cn (Y.M.); d202482140@hust.edu.cn (Y.Q.); m202476483@hust.edu.cn (Y.L.); 2 Department of Intensive Care Medicine, Wuhan Hospital of Traditional Chinese Medicine, Wuhan 430050, China; zjwh2008@hbucm.edu.cn

**Keywords:** cardiovascular disease, neutrophils, neutrophil extracellular traps

## Abstract

Cardiovascular disease (CVD) is currently recognized as one of the leading health threats to humanity. Neutrophils play an important role in innate immune response. The activation of neutrophils leads to the release of neutrophil extracellular traps (NETs) in response to various stimuli. Appropriate NETs are essential for maintaining homeostasis in the body, while excessive NETs will cause pathological damage. Accumulating evidence indicates that NETs are implicated in CVD pathophysiology. This review aims to provide a comprehensive review of the characteristics, signaling pathways, and interactions of NETs with other immune cells, and the comparisons of NETosis with other cell deaths, focusing on the role of NETs in CVDs. Furthermore, this study provides a theoretical basis for further improvement in targeted NET therapy for CVD.

## 1. Introduction

Cardiovascular disease (CVD) is the leading cause of death globally with a morbidity of youth-oriented tendency over the years, increasing healthcare and financial burden on society. Immune response and inflammation are involved in the occurrence and progression of CVD, manifesting as the sterile inflammation with infiltration of immune cells into lesions. Therefore, the role of the immune system in cardiovascular injury has received increasing attention. Immune cells have recently been in the spotlight of “Cardioprotection Beyond the Cardiomyocyte” [1]. Emerging therapeutic strategies that modulate the immune system are actively being developed for treating CVD [1]. During CVD, the initial response and migration of neutrophils to the “diseased” heart represent the earliest circulating cellular event [2], and numerous studies have been conducted on the role of neutrophils in CVD. Targeting neutrophils is a new potential therapeutic strategy for CVD.

Neutrophils are an essential component of innate immune cells, accounting for 40–70% of all circulating leukocytes, and serve as the initial defense against invading pathogens [3]. Neutrophils possess a broad spectrum of weaponry to eliminate intracellular and extracellular microorganisms, including phagocytosis, degranulation, and the release of neutrophil extracellular traps (NETs) [4]. NETs have recently attracted considerable attention. Brinkmann et al. indicated that NET forms as a novel killing form for extracellular bacteria by pathogens trapping with activated neutrophils in response to inflammatory stimuli [5]. NET is a reticular structure composed of nucleic acids, histones, and antimicrobial proteins, and their formation is termed NETosis [6]. During inflammation, neutrophils are activated, which is followed by further chromatin condensation, nuclear membrane disintegration, and plasma membrane disappearance. Then, nuclear contents are discharged into the cytoplasm, and the mixed nuclear proteins, granule proteins, and DNA are released outside the cells, marking the formation of NETs [7]. In contrast to the “suicidal NETosis,” there is another category of NETosis described as “vital NETosis,” which retains the structural integrity of neutrophils with further functions after NETs are released in the forms of vesicles [8]. Although NETs play an important role in eliminating microorganism elimination, excessive NETs caused by over-activation or reduced clearance may exacerbate disease progression.

Several studies have demonstrated that NETs play a pivotal role in CVD pathophysiology, including atherosclerosis [9], thrombosis [10], and multiple types of coronary artery disease (CAD) [11]. Basic research has established that targeting NETs can significantly improve CVD outcomes, offering promising new directions for therapeutic development [1]. Given the dominant role of NETs in CVD, this review summarizes extensive findings on their pathological mechanisms and therapeutic implications, and provides a comprehensive overview of this emerging field.

## 2. Characteristics and Functions of NETs

Since Brinkmann’s groundbreaking work, the composition and formation mechanisms of NETs have been the focus of intense research, but extensive studies are still lacking. The precise triggers that initiate NET formation remain debatable and controversial.

**Figure 1:**
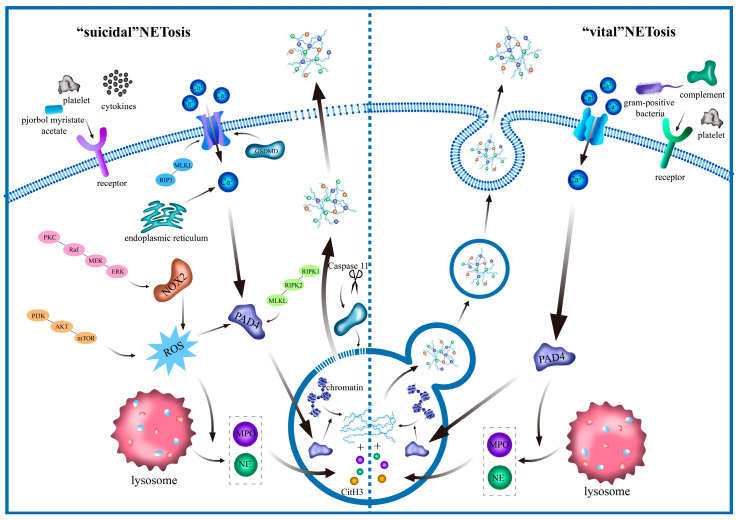
Mechanisms of NET formation. Two types of NET formation process (NETosis): “suicidal” and “vital.” Various materials include PMA, cytokines, and platelets, and can induce “suicidal” NETosis. Then kinase signaling cascades are activated, followed by a sequence of reactions including the deconstruction and release of chromatin into the cytoplasm, resulting in the interweaving of cytoplasm and karyoplasm; then NETs are released into the extracellular space, where NETs can exert their biological effects. Another kind of NETosis is called “vital” NETosis, which can be activated by platelets, complement proteins, and Gram-positive bacteria. In this process, after the materials are assembled in the nucleus, transport vesicles containing NETs are formed and germinated into the extracellular space without destroying the plasma membrane.

Nucleosomes are the basic units of chromatin in eukaryotic nuclei and are composed of histones and DNA. Histones bind to DNA via electrostatic interactions under physiological conditions. Citrullination of histone reduce their positive charge, weakens the histone-DNA affinity, and promotes chromatin decondensation. Peptidylarginine deiminase 4 (PAD4) mediates histone hypercitrullination, and citrullinated histone H3 (CitH3) is a marker of NET formation [12] (Figure 1). Previous studies have demonstrated that PAD4-deficient mice develop impaired NETs [13].

PAD4 is a calcium ion (Ca^2+^)-dependent enzyme that usually requires a high Ca^2+^ concentration to be activated in vitro [14]. Consequently, extracellular and intracellular Ca^2+^ pools are crucial factors in NETosis [15]. During NETosis, neutrophil activation usually increases the Ca^2+^ concentration. The mechanical stimulation of the endoplasmic reticulum induces the release of Ca^2+^, thereby elevating intracellular Ca^2+^ concentration and activating PAD4 [16,17]. In apoptotic neutrophils, caspase cleavage of gasdermin E promotes the formation of pores in the plasma membrane or in other intracellular compartments, accumulating Ca^2+^ within the cell and activating PAD4 (Figure 1), thereby triggering the NETosis process [18]. In 2015, David et al. reported that Ca^2+^ ionophore, A23187-activated nicotinamide adenine dinucleotide phosphate (NADPH) oxidase (NOX)-independent NETosis is fast and mediated by a Ca^2+^-activated small conductance potassium (SK) channel member SK3 and mitochondrial reactive oxygen species (ROS) [19]. Later, D’Cruz et al. demonstrated that neutrophils can co-opt the mixed lineage kinase domain-like (MLKL)-dependent pathway, in combination with PAD4 [20]. Receptor-interacting protein 3 (RIP3) phosphorylates MLKL, leading to its oligomerization and translocation to the plasma membrane [21] (Figure 1). Upon the localization of MLKL within the plasma membrane, a cascade of events unfolds, culminating in the formation of permeable ion channels. Ultimately, this intricate process elevates intracellular Ca^2+^ concentration [22]. Gasdermin D (GSDMD) is required for NETosis, and neutrophils lacking Caspase11 and GSDMD cannot citrullinate histones or form NETs [23]. Sollberger et al. reported that GSDMD alters cellular ion gradients by assembling pores on the plasma membrane, potentially facilitating the activation of PAD4 [24]. Another study demonstrated that neutrophils with mitochondrial Ca^2+^ uptake deficiency accumulated more Ca^2+^, augmenting suicidal NETosis [25]. In conclusion, the intracellular Ca^2+^ concentration serves as a pivotal trigger for NETosis, orchestrating this critical immune response.

Additionally, ROS plays a critical role in initiating NETosis and activating PAD4. In 2007, overgenerated ROS was an absolute requisite for inducing NETosis [26]. Upon stimulation, neutrophils produce ROS through NOX2 [27]. NOX inhibition disrupts NET formation [28]. ROS further facilitates the translocation of neutrophil elastase (NE) and myeloperoxidase (MPO) into the nucleus, where they degrade histones and promote chromatin decondensation [29]. In conclusion, NET release involves a series of events, including Ca^2+^ flux, kinase activation, ROS generation, and chromatin deconstruction (Figure 1) [26], ultimately leading to the extrusion of NETs into the extracellular space to perform their biological functions.

Platelets [16], complement proteins [16], and Gram-positive bacteria, such as *Staphylococcus aureus* [30], tend to activate “vital” NETosis. Upon stimulation, neutrophils facilitate Ca^2+^ influx via SK3 channels, orchestrating a critical step in their activation process [19]. Elevation of Ca^2+^ concentration triggers activation of the PAD4 enzyme, resulting in CitH3 formation and chromatin decondensation. In this process, the inner and outer membranes of the nucleus separate after the early decondensation of the nuclear material. Subsequently, transport vesicles containing nuclear DNA are formed and germinate in the extracellular space via the plasma membrane. The entire process takes approximately 30 min without destroying the plasma membrane with rapid kinetics (5–60 min) [31], which is different from suicide NETosis (Figure 1).

## 3. NET Signaling Pathway

NETs play a crucial role in tissue repair and defense and are regulated by multiple signaling pathways.

The mitogen-activated protein kinase (MAPK) signaling pathway enhances NET formation by activating NADPH oxidase and anti-apoptotic proteins; this process can be inhibited by compounds such GW5074 and U0126 [32,33]. Besides the MAPK pathway, other signaling pathways, including nonreceptor tyrosine kinase Janus kinase (JAK) 2 [34], spleen tyrosine kinase (SYK)-Phosphatidylinositol 3-kinase (PI3K)-mammalian target of rapamycin complex (mTORC)2 [35,36,37], nuclear factor kappa-B (NF-κB) [38], and receptor-interacting protein kinase (RIPK)1/RIPK3/MLKL-PAD4, are involved in regulating NET release [20].

Additionally, researchers have found that NETs respond to multiple signaling pathways and actively participate in the activating these signaling pathways and influencing disease progression. In acute brain injury, NETs exacerbate tissue damage via the STING-IRE1α/ASK1/JNK pathway, which can be mitigated by PAD4 inhibition [39]. NETs have also been demonstrated to contribute to cerebrovascular complications caused by tissue plasminogen activator (tPA) by activating the cGAS-STING pathway and including a type 1 interferon (IFN) response in the ischemic brain [40]. NETs impair wound healing under diabetic conditions by suppressing the toll-like receptor 9 (TLR9)-p21-activated kinase 2 (PAK2)-dependent Hippo-YAP signaling pathway [41] and development of atherosclerosis by inducing TLR9/NF-κB-mediated interleukin-8 (IL-8) secretion in macrophages [42].

The precise mechanisms by which NETs contribute to disease pathogenesis are not yet fully understood, highlighting the need for further investigation of their regulatory pathways to uncover potential therapeutic strategies.

## 4. Interaction of NETs with Other Immune Cells

**Figure 2:**
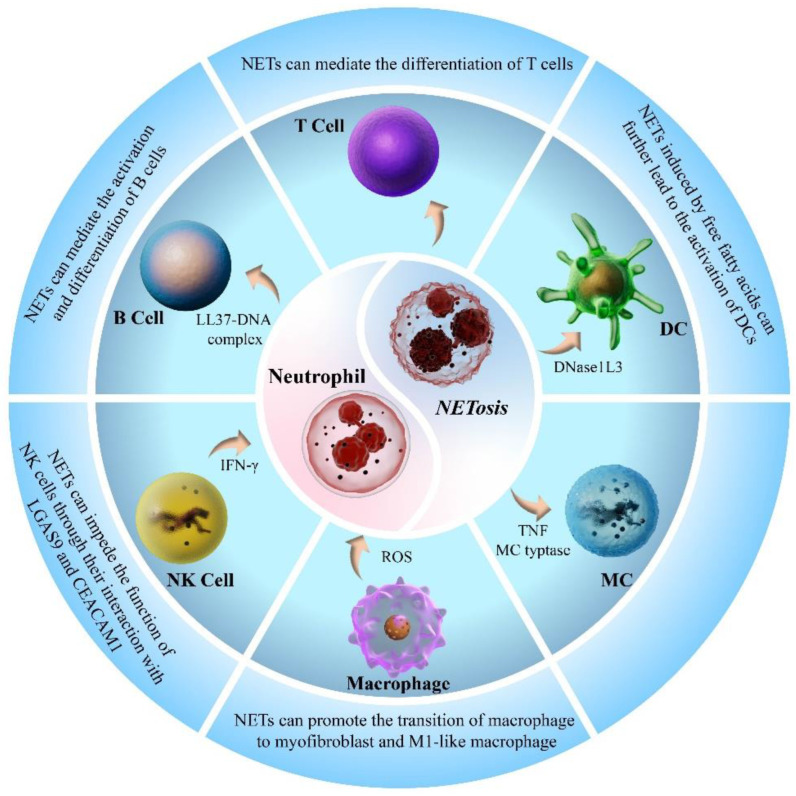
NET interaction with other immune cells. Overview of the connections between NETs and other immune cells. Present the important role of NETs in the immune system.

Neutrophils, as the vanguard of the immune system’s defense against pathogen invasion, are typically the first responders recruited to the site of inflammation. Additionally, neutrophils play a crucial role in orchestrating the immune response by interacting with other immune cells and contributing to disease initiation and progression. Mounting evidence suggests that NETs serve as a critical mechanism by which neutrophils engage with and modulate the activity of other immune cells (Figure 2).

During the immune response, T cells serve as the primary defense against the invasion of pathogenic microorganisms. Recent studies have indicated that NETs can effect T-cell differentiation [43,44,45,46,47,48].

NET also mediates B-cell differentiation [49,50]. Gestermann et al. confirmed that the LL37-DNA complex of NETs could enter the endosomal compartments of B cells to activate memory B cells (Figure 2) [51]. Another study demonstrated that NETs can drive B-cell differentiation into plasma cells by activating the MAPK cascade [52].

Natural killer (NK) cells are the primary effectors of almost all the immune functions within the lymphatic system. They contribute to thrombosis by promoting NET formation via type II interferon γ (IFN-γ) secretion (Figure 2) [53]. NETs may suppress NK cell activity via interactions between LGAS9 and CEACAM1. LGAS9 may also regulate PAD4 expression [54]. Furthermore, cathepsin G, a predominant component of NETs [55], compromised NKp46-mediated NK cell activation [56].

Dendritic cells (DCs) may play a central role in initiating, orchestrating, and sustaining the immune response. Scientists have discovered that NETs induced by free fatty acids can further activate DCs [46], indicating potential associations between DCs and NETs. Moreover, the release of DNase1L3 from DCs plays a crucial role in the extracellular degradation of NETs [57] (Figure 2).

Mast cells (MCs) are tissue-resident cells that secrete various cytokines to drive allergic reactions and serve as key mediators for the recruitment of neutrophils [58]. The major component of MC-secreting granules, MC tryptase, enhances the formation of PMA-induced NETs [59]. Additionally, evidence suggests that tumor necrosis factor (TNF) facilitates NET formation [60,61]. Dudeck et al. demonstrated that TNF released by MCs can directly activate circulating neutrophils via TNF receptors on the neutrophil surface [58], indicating the potential role of MCs in regulating NET formation via TNF (Figure 2).

Macrophages are closely associated with NETs and can facilitate their formation via the ROS/GSDMD axis [62]. Therefore, NETs can induce macrophage polarization toward the M1 phenotype [63,64]. Moreover, macrophages contribute to the degradation and clearance of NETs via DNase-mediated mechanisms [65,66]. Recent studies have highlighted the bidirectional interaction between macrophages and NETs, which plays a crucial role in regulation immune response.

## 5. Distinction Between NETosis and Other Cell Deaths

In Branklin’s Prototype mode, NETosis differs from apoptosis or necrosis. Additionally, pharmacologically, the various modes of cell death require distinct agonists [67,68,69] and inhibitors [70,71,72,73] (Table 1).

Apoptosis, first identified in 1972 [76], also called programmed cell death is a form of programmed cell death characterized by membrane blebbing, decreased cell size, and apoptotic body formation with an intact plasma membrane [77]. During apoptosis, cytochrome C is released from the mitochondria [74] and DNA undergoes internucleosomal fragmentation [78]. In contrast, NETosis involves the destruction of the nucleus and plasma membrane, while preserving DNA integrity to form the structure of NETs [79].

In 2012, Dixon et al. first identified ferroptosis, an iron-dependent form of programmed cell death that differs from apoptosis and necrosis [80]. It is triggered by glutathione depletion and the reduce activity of glutathione peroxidase 4 (GPX4), leading to lipid peroxidation and ROS accumulation [75]. Morphologically, it involves mitochondrial shrinkage, dense mitochondrial membranes, reduced cristage, and rupture of the outer mitochondrial membrane. Ferroptosis also features cell membrane fragmentation, similar to that observed in NETosis. Due to the central role of ROS in both processes, the connection between ferroptosis and NETosis has gained increasing scientific attention.

Pyroptosis has been recognized as a unique form of programmed cell death since its discovery in 1992. It follows a classical pathway involving caspase-1 activation, which cleaves GSDMD, leading to cell membrane perforation. The resulting membrane pores allow for the release of inflammatory factors and disrupt cellular control over substance transport, ultimately triggering pyroptosis. Research has also revealed that GSDMD participates in NET formation, and NETs significantly influence pyroptotic. This link between NETosis and pyroptosis has been observed in various cell types, including macrophages [81], neurons [82], etc.

## 6. The Roles of NETs in CVDs

### 6.1. NETs in Thrombosis

Ischemic heart disease and stroke were the leading thromboembolic disease that caused disability and death in 2019 [83]. Thrombosis, which is usually composed of red blood cells, platelets, and insoluble fibrin, is the pathological basis of myocardial infarction (MI), cerebral stoke, and venous thromboembolism (VTE), and has received extensive attention clinically. When a thrombus develops, it can impede or completely obstruct the blood flow, dislodge, and migrate to other vital organs, leading to potentially catastrophic outcomes.

Emerging evidence has indicated the complexity of thrombosis. Recent findings suggest that NETs contribute to thrombosis, a phenomenon that has not been fully recognized yet. Perdomo et al. found that NETosis is essential for thrombus formation in heparin-induced thrombocytopenia, and the inhibition of NETs could be exploited therapeutically [84]. Similarly, NETs are also involved in venous thrombosis and arterial thrombosis.

Thrombosis can be induced by the initiating a coagulation cascade that is divided into intrinsic and extrinsic pathways. Recent studies have demonstrated that NETs can directly stimulate the coagulation cascades. Tissue factor (TF) is a major mediator of thrombosis and is vital for the primary activation of coagulation [85]. Stakos et al. demonstrated that NETs can expose functional TF, leading to platelet activation and thrombin generation [86]. NETs can initiate FXII-dependent coagulation, thereby establishing a cascade of events that culminate in thrombus formation. Von Brühl et al. suggested that NETs can bind to FXII and facilitate the activation of FXII. Negatively charged surfaces are reported to contribute to FXII activation; therefore, NETs may activate FXII via their negatively charged extracellular DNA surfaces [87].

Recently, researchers found that NETs are involved in the initiation of complement activation. Bryan et al. found that *C3^−/−^* mice (lacking complement component C3) could not release histones or nuclear DNA, indicating that NET formation is highly dependent on C3 [30]. Guglietta et al. demonstrated that C3a-receptor deficient mice lost the ability to form NETs [88]. Ortiz-Espinosa et al. proposed that C5a and C5aR1 induce NETs in polymorphic mononuclear neutrophil (PMN)–myeloid-derived suppressor cells, and C5a or C5aR1 inhibition abrogates the formation of NETs [89]. However, in preliminary experiments, the C3a receptor played a greater role in NET production than the C5a receptor did. Additionally, properdin, factor B, and C3 are deposited on PMA-induced NETs. MPO, cathepsin G, and proteinase 3 can bind and activate properdin, indicating that NETs may form a platform for complement activation. Yuen et al. stimulated neutrophils with PMA and incubated them in a complement-competent buffer. After minutes, the terminal complement complex C5b-9 was deposited on NETs using immunofluorescence microscopy [90], and the application of DNAse for NET significantly decreases C5b-9 levels, indicating the necessity of NETs for activation of complement cascade activation and terminal complement complex deposition.

**Figure 3:**
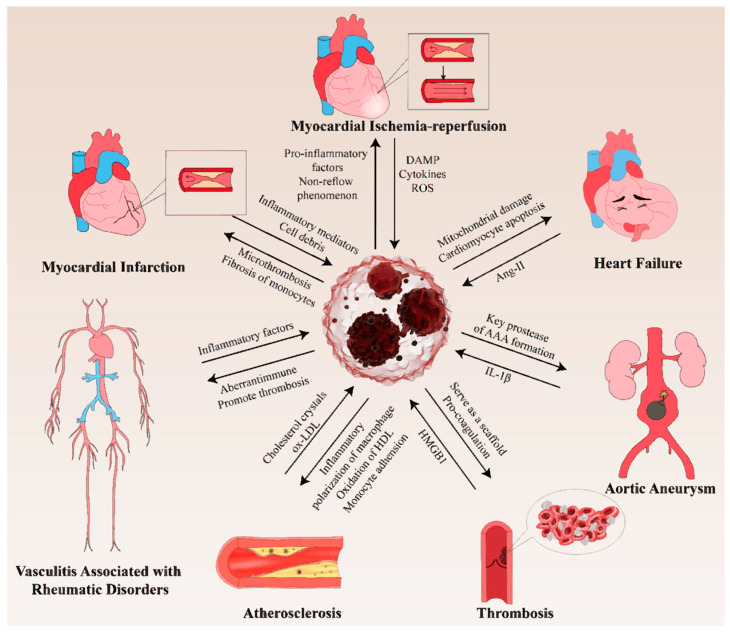
NETs in CVDs. NETs have been proven to be involved in pathophysiology of CVDs, including atherosclerosis, thrombosis, vasculitis, aortic aneurysm, and multiple types of coronary artery disease.

Platelets are essential components of hemostasis and thrombosis. Mounting evidence suggests that platelets may play a pivotal role in NET formation [91]. Neutrophils recognize CD62-P (known as P-selectin) via PSGL-1 [92], a receptor on activated platelet alpha granules, thereby establishing a connection between neutrophils and platelets. This phenomenon has been demonstrated to facilitate the formation of NET. However, antibodies that block P-selectin do not interfere with NET production elicited by activated platelets [93], indicating that other mechanisms of their interaction induce NETosis. Further research has proven that High Mobility Group Box (HMGB)1 secreted by platelets facilitates NET formation. Maugeri et al. discovered that *Hmgb1^−/−^* platelets failed to elicit NETs, whereas the HMGB1 alone was committed to NET generation by neutrophils, indicating that activated platelets present HMGB1 to drive NET generation [93] (Figure 3).

Chilingaryan et al. discovered the potential involvement of NETs induced by erythrocytes through activation platelets in coronary arterial thrombosis. Strongly expressed CitH3 were observed in all human thrombi, confirming that NETs are a common component of coronary thrombosis [94]. Additionally, a strong NET signal was co-located with areas of red blood cell (RBC) accumulation in their study, suggesting that NETs “capture” RBCs, which constitutes the majority of coronary artery thrombus.

In summary, NETs orchestrate the activation of white blood cells, platelets, and endothelial cells, thereby eliciting robust pro-inflammatory responses that culminate in endothelial dysfunction. Furthermore, the intricate network structure of NETs serves as a scaffold for thrombus formation. Additionally, NETs facilitate thrombosis by engaging in complex interactions with coagulation factors, complement factors, platelets, endothelial cells, and RBCs (Figure 3).

### 6.2. NETs in Atherosclerosis

Atherosclerosis is widely acknowledged as a chronic inflammatory and lipid-driven disease that is the foremost cause of CAD, carotid artery disease, and peripheral arterial disease within the cardiovascular system. The compromised or diseased intima frequently serves as the primary instigator of this condition. In response, inflammatory cells are summoned to the site of injury, adhering to the vessel wall, fostering plaque formation, progressively narrowing the vessel lumen, and hindering blood flow. As the pathology advances, plaque rupture may ensue, further exacerbating the detrimental effects and precipitating more severe pathological damage.

Megens et al. pioneered the discovery of NETs in atherosclerotic lesions in murine models and humans [95]. Subsequently, several human studies have demonstrated a positive correlation between circulating NET markers and the extent of atherosclerotic lesions. [96]. In 2015, Warnatsch et al. established ApoE/PR3/NE-deficient mice to block NETosis in vivo. Following an eight-week regimen of a high-fat diet administration, these genetically modified mice exhibited a threefold reduction in plaque size compared to their ApoE-deficient counterparts. This significant diminution was corroborated by en face analysis of intact aortas, underscoring the pivotal role of NETs in the atherosclerosis pathogenesis [97]. They also found that cholesterol crystals (CCs), a regular feature within the necrotic core of atherosclerotic plaques, could induce NETosis [98]. Furthermore, in a separate study, the eliminating NETs in the plaques of diabetic mice using DNase-I significantly enhanced the regression of atherosclerosis compared to control mice [63]. This finding suggests that targeting NETs is a promising therapeutic strategy for treating patients with diabetes and atherosclerosis.

Basic and clinical research data have proven that a series of pathophysiological changes caused by oxidized low-density lipoprotein (ox-LDL) are the key to the formation of atherosclerosis and are closely related to the severity of atherosclerosis [99]. The ox-LDL can cause NET release, which is attributed to the signaling mechanism of TLR2/6 and the PKC-IRAK-MAPK pathway, as elucidated by Awasthi et al. [100]. Another study displayed that PMA-induced NET formation was significantly enhanced by additional incubation with ox-LDL in HL-60-derived neutrophils [101]. Furthermore, ox-LDL can decrease the expression of CFTR, a chloride ion channel protein, elevating the intracellular chloride ion concentration and facilitating NET formation [102].

High-density lipoprotein (HDL) is renowned for its vascular protective properties. However, when apolipoprotein A-I (Apo A-I), a predominant protein in HDL, is oxidized by chlorination or nitration of tyrosine residues (Cl-Tyr and n-Tyr oxidizing HDL, respectively), the vasoprotective ability of the lipoprotein is weakened while it gains pro-inflammatory activity [103]. In a study on systemic lupus erythematosus, MPO produced during NETosis was demonstrated to contribute to the oxidation of HDL, and NET inhibition effectively reduced the levels of pro-inflammatory n-Tyr-oxidized HDL in vivo [103]. NETs facilitate the oxidation of HDL, thereby hastening the development of atherosclerotic plaques. However, further studies are required to clarify the interactions between NETs and HDL.

NETs can also react in other ways to atherosclerosis. Studies have demonstrated that NETs accelerate atherosclerosis during endotoxemia by mediating charge-dependent monocyte adhesion to NETs [104]. Additionally, as mentioned above, NETs are involved in atherosclerosis through by promoting the polarization of macrophages towards inflammatory phenotypes. For the first time, the latest research has demonstrated that using high-dose statins in patients with CAD can effectively reduce NET formation, thereby reducing inflammation and providing a theoretical basis for using statins at appropriate doses [105]. In conclusion, targeting NETs may offer a potential approach for the clinical amelioration of atherosclerosis (Figure 3).

### 6.3. NETs in MI

Acute myocardial infarction (AMI) is a devastating condition characterized by myocardial, resulting from acute and sustained ischemia and hypoxia in the coronary arteries. It is the leading cause of mortality worldwide. AMI predominantly arises in the backdrop of coronary atherosclerosis, typically manifesting as lumen occlusion due to thrombosis following the rupture of an atherosclerotic plaque. Moreover, coronary artery spasm also plays a significant role as a hidden culprit in heart attacks. The involvement of inflammation in the CVD progression was first supported by the Physician’s Health Study [96]. During ischemic injury, many neutrophils are attracted to inflammatory mediators and cell debris and are recruited into the injured tissue [64].

Microthrombosis has been reported to be associated with cardiac microvascular dysfunction or disturbances with AMI [106]. NETs play an important role in thrombosis, indicating their involvement in MI.

NETs participate in the formation of ventricular aneurysms after MI [107]. Another study demonstrated that NETs were associated with ST-elevation myocardial infarction (STEMI) via bootstrap sensitivity analysis [108]. Increased levels of NET markers and highly activated fibrocytes have been detected in regions of myocardial ischemia. An in vitro experiment demonstrated that NETs induce differentiation of monocytes into fibrocytes [109] (Figure 3), suggesting that NETs may be involved in myocardial tissue remodeling after STEMI. Consequently, it is reasonable to hypothesize that targeting NETs may affect the fibrosis and improve AMI prognosis.

However, the role of NETs inhibition in MI remains debatable. In 2019, Du et al. were the first to report that inhibiting PAD4, the essential enzyme for driving NETosis, by GSK484 exerts a protective effect on MI. Their findings revealed that GSK484 significantly reduced infarct size and serum myocardial enzyme concentrations. Simultaneously, it downregulates the expression of pro-inflammatory cytokines, including IL-1β, IL-6, and TNF-α, thereby mitigating the MI-induced apoptosis of myocardial cells [110]. In contrast, in the same year, NETs were reported to promote the polarization of macrophage toward a reparative phenotype after MI, restrain the pro-inflammatory macrophages under hypoxia, and reduce the expression of IL-6 and TNF-α expression in vitro research [111]. Therefore, future research endeavors must delve deeper and embrace a more comprehensive approach.

### 6.4. NETs in Myocardial Ischemia–Reperfusion (MI/R)

Although thrombolytic therapy or primary percutaneous coronary intervention (PCI) can effectively and promptly achieve myocardial reperfusion to ameliorate acute myocardial ischemic injury [112], an increasing body of research has revealed that unpredictable reperfusion injuries often accompany these treatments. This unforeseen damage can further exacerbate myocardial necrosis in the infarct area and significantly worsen clinical outcomes. Studies have indicated that NETs are also associated with the occurrence of ischemia–reperfusion disease.

In 2015, Ge et al. demonstrated the existence of NETs in I/R-challenged myocardium [113]. Pashevin et al. investigated the effects of co-culturing neonatal rat cardiomyocytes with PMNs, established a hypoxia–reoxygenation model, and found that proteasome inhibition prevents NET-induced cardiomyocyte death [114]. New research has demonstrated that inhibition of C5a-C5aR1 axis reduces NET formation and alleviates myocardial I/R injury [11].

NETs may further exacerbate MI/R injury by upregulating the expression of pro-inflammatory factors, thereby amplifying the inflammatory response and aggravating tissue damage. Considerable research has demonstrated that extracellular histone released during NETosis is pro-inflammatory, and they can bind and activate TLR2/4/9 on innate immune cells, thereby initiating the MyD88-dependent NLRP3 inflammasome signaling pathway, releasing downstream pro-inflammatory factors, and establishing a sterile inflammatory environment, ultimately inducing myocardial damage [115]. Meanwhile, histones can also function as damage-associated molecular patterns, promoting the release of NETs and aggravating injury. These secreted proteins include HMGB1 and LL37 from NETs [116]. All this evidence suggests that the pro-inflammatory effect of NETs contributes to ischemia–reperfusion injury.

The phenomenon of no-reflow has been reported to underlie the occurrence of reperfusion injury, contributing significantly to its pathophysiology. Shao et al. indicated that microthrombosis is involved in the occurrence of cardiac non-reflow phenomenon in rats with acute MI/R [106]. Ge et al. elucidated the mechanism by which NET-induced microthrombosis in I/R leads to the no-reflow phenomenon and explored the therapeutic strategies based on DNAse-targeting NETs that can be used to ameliorate the no-reflow phenomenon after cardiac I/R injury [113].

In summary, ischemia reperfusion injury can release ROS and upregulate cytokines, which are vital for the NET formation. Accordingly, NETs exacerbate IR injury by engaging its primary components with cytokines, thereby fueling inflammation and microthrombosis and creating a vicious cycle that amplifies tissue damage (Figure 3).

### 6.5. NETs in Heart Failure (HF)

HF is a clinical syndrome caused by impaired cardiac pumping function that prevents the heart from meeting the body’s metabolic needs [117]. It can result from acute cardiac events or progressive chronic damage [118]. HF involves complex pathogenic mechanisms that are often associated with chronic inflammation. Growing evidence indicates that NETs promote HF progression by intensifying inflammation [119].

Based on the measurement of left ventricular ejection fraction, HF is categorized into three types: HF with Reduced Ejection Fraction (HFrEF), HF with Preserved Ejection Fraction (HFpEF), and HF with Mid-Range Ejection Fraction (HFmrEF), based on the measurement of left ventricular ejection fraction [120]. Benjamin et al. found that systemic NET biomarkers and inflammatory inducers in the blood of patients with ADHFpEF were significantly elevated and positively correlated with circulating low-density neutrophil (LDN) counts [121]. Another clinical plasma analysis revealed higher levels of the NET marker MPO in patients with HFpEF than in healthy controls, indicating that MPO-dependent oxidative stress contributes to HFpEF progression [122]. In 2025, transcriptomic analysis enabled a more accurate differentiation between HFrEF and HFpEF, further supporting the role of NETs in accelerating HFpEF and their potential as a therapeutic targets [123]. Research on HFrEF has also advanced: a cross-sectional study by Vibeke et al. confirmed that NETs promote HF progression in HFrEF, with CITH3 levels linked to disease severity [124]. Another study found that MPO was associated with impaired left ventricular diastolic function and right ventricular systolic function in patients with HFrEF [125].

In 2024, scientists used endomyocardial biopsy samples to confirm the clinical relevance and prognostic value of NETs in HF and dilated cardiomyopathy for the first time. These findings suggested that NETs may worsen cardiac dysfunction by causing mitochondrial damage [126]. Xudong et al. identified four NET-related diagnostic biomarkers, CXCR2, FCGR3B, VNN3, and FPR2, using GEO analysis to predict HF risk. These findings were validated in transverse aortic constriction (TAC) mice [127].

In addition to studies based on clinical samples, numerous researchers have explored the impact of NETs on HF by constructing HF models in animals. Researchers have found that, in TAC-induced HF mouse models, NETs trigger cardiomyocyte apoptosis and mitochondrial dysfunction, impairing mitochondrial biological processes. Moreover, sacubitril/valsartan, commonly used to treat HFrEF, can inhibit NETosis via the VWF-SLC44A2-NET axis and improve HF prognosis [128]. DEL-1 is an anti-inflammatory glycoprotein that has been proven to exacerbate HF injury by increasing NET production [129]. In an Ang-II-induced non-ischemic HF model, researchers revealed that NETs mediate the response of the heart to Ang-II. Ang-II activates neutrophils, causing them to adhere to the blood vessel walls and release NETs, thereby causing microvascular thrombosis and worsening myocardial hypoxia [117]. In the same model, KLF2, a potent repressor of myeloid proinflammatory activation [130], may further exacerbate HF by suppressing the release of more NETs [131].

Left ventricular assist devices (LVADs) are frequently used in clinical practice as therapeutic interventions for patients with end-stage HF. A study comparing the levels of CITH3-NET markers in the serum of patients with HF before and after LVAD implantation found that NET levels dropped below the baseline [119]. Tomasz et al. also verified this perspective. They found that the abnormal increase in citH3 was associated with the expansion of the right ventricular diameter in some patients after LVAD surgery, and they believed that the abnormal changes in citH3 could be regarded as an early possible marker of an increased risk of right ventricular failure [132].

Overall, NET levels are elevated and contribute to disease progression in patients with HF, especially those with HFpEF. Inhibiting NET formation using targeted therapies may significantly reduce HF severity and improve clinical outcomes. Additionally, LVAD, a common treatment for advanced HF, reduce NET formation and provides therapeutic benefits.

### 6.6. NETs in Vasculitis-Associated Rheumatic Disorders

There is increasing evidence for the central role of neutrophils in the vasculitic responses. Antineutrophil cytoplasmic antibody (ANCA)-associated vasculitis (AAV) is an autoimmune disease involving multiple systems throughout the body that is associated with the alternative complement pathway (AP). The ANCA-mediated activation of neutrophils has been proven to induce NET formation. Additionally, proteinase-3 and MPO, the components of NETs, as auto-antigen of ANCA, can continuously worsen the immune response of AAV patients [133]. In 2017, another study demonstrated that ANCA induces NETs via RIPK 1/3- and MLKL-dependent necroptosis, and the formed NETs further cause endothelial cell damage and provide a scaffold for AP activation, thereby aggravating AAV [134].

Neutrophils produce and release adenosine at the site of inflammation, which can regulate various functions of neutrophils in turn. Deficiency of adenosine deaminase 2 (DADA2) is a recessively inherited autoinflammatory disease caused by a functional loss or mutation of the ADA2 gene, resulting in systemic vasculitis. In 2019, Carmona-Rivera et al. first reported enhancement of extracellular adenosine-mediated NET formation in DADA2. They found a significant reduction in NET formation with inhibition of NOX or PADs after adenosine stimulation, indicating that adenosine induces NETosis via NOX- and PAD-dependent pathways in DADA2 pathogenesis [135].

Weckbach et al. reported the presence of NETs in the cardiac tissue of patients and mice with myocarditis. NETs promote cardiac inflammation in a mouse model of experimental autoimmune myocarditis (EAM). Tnd blocking of cytokine MK reduce NET^+^ PMNs, suggesting that NETs may play a role in EAM progression through cytokine MK [136].

Antiphospholipid syndrome (APS), also known as Hoghes syndrome, clinically manifested as recurrent thrombotic events and miscarriages. Given the well-established link between NETosis and thrombosis, we hypothesized that NETs may play a significant role in APS pathogenesis. It has been demonstrated that neutrophils in patients with APS are apt to overgenerate NETs, which is attributed to the involvement of human antiphospholipid antibodies and monoclonal antibodies, and research also suggest that these circulating NETs contribute to thrombotic events [137,138]. Ali et al. demonstrated the mechanism by which defibrotide inhibits neutrophil-mediated thrombotic inflammatory responses in APD for the first time [139], after clarifying the important role of NETs in APS. They identified that defibrotide could prevent disease-relevant NET formation, thereby hindering APS progression.

In systemic lupus erythematosus (SLE), neutrophils, especially NETs, have been demonstrated to play a pivotal role both in the initiation and maintenance of the aberrant immune responses, including the development of organ damage and vasculopathy [140] (Figure 3). In 2016, Dieker et al. elucidated the presence of apoptotic cell-derived microparticles within circulating microparticles of patients with SLE, which exert pro-inflammatory effects on plasmacytoid DCs and myeloid DCs while enhancing NETosis [141]. In rheumatoid arthritis (RA), autoantibodies and inflammatory cytokines induce NETosis, thereby stimulating the activation and release of inflammatory cytokines from fibroblast-like synoviocytes [142], which are crucial in RA pathogenesis [143]. The indicates that joint damage and NETs form an amplifying loop that aggravates RA progression.

### 6.7. NETs in Aortic Aneurysm

An aortic aneurysm is characterized by a pathological dilation of the aorta (>50% of its normal diameter) and presents a significant risk of mortality. The most prevalent form is AAA. Inflammation has recently emerged as a pivotal factor in AAA development, with circulating neutrophils playing a dominant role in the early stages.

Studies have revealed that NETs are essential drivers of AAA (Figure 3). NET components, such as histone citrullination, are abundant in the serum and aortic tissues of patients with AAA [144], and NETs exhibit a significant predictive value in disease prognosis, progression rate, and fracture risk assessment. Additionally, NE, the main component of NETs, is a key protease that promotes AAA formation. Yan et al. reported that neutrophil protease-mediated NET release contributes to the elastase-induced AAA model by promoting plasmacytoid dendritic cell (pDC) activation and type I IFN production [145]. In 2018, Meher et al. detected the colocalization of NETs with IL-1β in human AAAs. Further research has confirmed that IL-1β can trigger ceramide synthesis in aortic infiltrated neutrophils, resulting in NETosis, leading to the sequential formation of AAA [146] (Figure 3). Another study demonstrated that activated neutrophils in AAA can be stimulated to produce NETs by oxLDL, thereby exacerbating disease progression [147].

Increasing evidence indicated that inhibition of neutrophil migration and NET formation can alleviate AAA. Wei et al. reported that YW3-56, a PAD4 inhibitor, significantly alleviated Ang II-induced AAA rupture by blocking NETs formation [148]. Chen et al. demonstrated that NETs promote AAA formation by inducing ferroptosis in smooth muscle cells (SMCs) by inhibiting the PI3K/AKT pathway. Additionally, mesenchymal stem cell-derived extracellular vesicles (MSC-EVs) can protect against Ang II-induced AAA by inhibiting the NETosis-related pathway [149]. In another study, NETs have been proven to induce AAA formation by initiating intracellular signaling pathways via TLR-9 and subsequently promote the pro-inflammatory SMC phenotype via the Hippo-YAP pathway [150]. Furthermore, researchers discovered that resolvin D1 diminishes NET formation by reducing the level of citrullination, thereby inhibiting the formation of AAA in elastase perfusion-treated mice model and Ang II-infused AAA models [151]. Based on the characteristics of the inflammatory response of AAA and the interaction between immune cells, NETs may be involved in AAA pathophysiology by interacting with other immune cells.

## 7. Potential Therapy Targeting NETs

As NETs are gradually gaining importance in basic research, pharmacological strategies aimed at targeting NETs are emerging as a promising frontiers across a spectrum of disease models. Erythromycin, a macrolide antibiotics, has been demonstrated to reduce the production of NETs in smoking-related chronic pulmonary inflammation [152]. A clinical trial revealed that rituximab and belimumab possess a remarkable ability to mitigate autoimmune phenomena in SLE by effectively suppressing the excessive formation of NETs [153]. In the previous section, we investigated the role of NETs in CVD pathogenesis. We demonstrated the effect of targeting different NET-related molecules to inhibit NET production in multiple CVD animal models. Consequently, we believe that the NET formation process and NET itself could serve as potential therapeutic targets for the clinical prevention and treatment of various CVDs.

Cytotoxic proteases, the primary component of NETs, play a pivotal role in eliminating pathogens, but may also cause injury to the surrounding tissue and cells in excess. Pieterse suggested that targeting NE in NETs may serve as a therapeutic strategy for managing vascular leakage. Inhibition of the physiological function of NETs alleviates their mediated vascular leakage and promotes fibrosis under inflammatory conditions [154]. DNase is a non-specific endonuclease that digests single or double-stranded DNA and can degrade NETs by destroying the skeleton structure formed by DNA. In a diabetic model, DNase treatment reduced NETs-induced plaque macrophage inflammation and promoted atherosclerosis regression in diabetic mice [63]. A recent retrospective observational study, leveraging a substantial cohort of clinical samples, demonstrated that adding DNase I to disaggregate components of NETs can enhance the efficacy of thrombolytic therapy and improve patient outcomes [155]. Chrysanthopoulou et al. indicated that the NETs component MPO plays a crucial role in fibroblast differentiation, and MPO inhibition reduces α-SMA expression in NET-treated lung fibroblasts, resulting in the inhibition of cell fibrosis [107].

Although numerous methods are available to detect NETs in basic research, the clinical detection of NETs is constrained by variations in sensitivity, specificity, and reproducibility [156]. Moreover, there is currently a lack of standardized diagnostic methods for NETs in human samples. According to a study in 2021, Hayden et al. reported that ELISA measurements for MPO-DNA detection lacked sufficient sensitivity in human plasma samples [157]. However, the developing of clinical studies pertaining on NET is imperative. Selective inhibition of NETs in the pathological state without interfering with their normal physiological function will become a breakthrough point for treating CVDs in the future.

## 8. Challenges and Future Directions

Neutrophils, as indispensable guardians of the body’s immune system, have been increasingly recognized for their pivotal role in orchestrating a myriad of cardiovascular events. NETs, which function as an impressive and sophisticated defense mechanism against a myriad of pathogens, have increasingly captured the attention of the scientific community in recent years. Although NETs play an important role in maintaining homeostasis, excessive NETs can cause pathological damage. As discussed above, NETs are indeed important and significantly affect the initiation and progression of CVDs, especially in the pathophysiology of thrombosis and atherosclerosis. Most functional studies have been conducted using mice and other animals. However, studies have demonstrated that the expression and characteristics of neutrophils vary significantly across different biological species. Accordingly, more clinical trials are needed to confirm the feasibility of targeting NETs for CVD treatment. In addition, NETs play a vital role as the first line of defense against invaders; therefore, long-term targeted therapy in chronic CVDs may come at the expense of weakening host defense, which is also an issue that cannot be ignored in the developing of related therapies.

In summary, achieving an optimal balance between preserving the reparative functions of neutrophils and mitigating their pro-inflammatory effects is paramount. This delicate equilibrium is crucial for identifying and refining the most appropriate molecular targets and ultimately paving the way for innovative and effective therapeutic approaches in cardiovascular medicine.

## Figures and Tables

**Table 1:** Major types of cell death.

Type	Morphological Features	Biochemical Features	Biomarker	Pharmacological Modulators	Regulator	Ref.
NETosis	The plasma membrane ruptures; NET with reticulate structure are released into the extracellular space	Intracellular calcium concentration; ROS production; Deconstruction of chromatin; Rupture of plasma membrane (suicide NETosis)	CitH3, MPO, NE, PAD4	activator: PMA inhibitor: Cl-amidine	positive: IL-8, LPS, activated platelets, TNF-α negative: metabolites released from apoptotic cells act as tissue	[39,42]
Apoptosis	Membrane blebbing; Decreased cell size; Formation of apoptotic body; Maintain integrity of organelle	Fragmentation of DNA; Activation of caspase cascade; Release of cytochrome c from mitochondria; Phosphatidylserine is everted	caspase-3, Bcl2, Bax, PARP	activator: Dexamethasone inhibitor: Emricasan, Z-VAD-FMK	positive: p53, Bax, TGF-β negative: Bcl-2, Bcd-XL, IL-4	[67,70,71,74]
Ferroptosis	Shrinking of mitochondria with compact membrane; Fewer mitochondrial ridges and outer mitochondrial membrane rupture	Depletion of glutathione; Lipid peroxidation; Increase in ROS	PTGS2, GPX4, ATG, ACSL4	activator: Sorafenib, Erastin inhibitor: Ferrostatin-1, Liproxstatin-1, Troglitazone, Rosiglitazone, Pioglitazone	positive: RSL3, RAS, p53 negative: GPX4, SLC7A11, FSP1, NRF2, DFO	[68,72,75]
Pyroptosis	Cells are swollen; Formation of ballooning bubbles; Perforation of cell membrane	Formation of inflammasomes; Activation of Caspase1 and GSDMD; Intense inflammation	caspase-1, caspase-4/5/11, GSDMD, IL-1β	activator: Polyphyllin VI inhibitor: Z-VAD-FMK, Q-VD-Oph	positive: GSDMD, NLRP3, Caspase-1, Caspase-11 negative: -	[69,71,73]

## Data Availability

Not applicable.

## Abbreviations

The following abbreviations are used in this manuscript:

AAA	abdominal aortic aneurysm
AV	antineutrophil cytoplasmic antibody-associated vasculitis
AKT	protein Kinase B
AMI	acute myocardial infarction
ANCA	antineutrophil cytoplasmic antibody
AP	alternative complement pathway
Apo A-I	apolipoprotein A-I
apoVs	apoptotic vesicles
APS	antiphospholipid syndrome
Ca^2+^	calcium ion
CAD	coronary artery disease
CC	cholesterol crystal
CitH3	citrullinated histone H3
CVD	cardiovascular disease
DADA2	deficiency of adenosine deaminase 2
DC	dendritic cell
DNase I	deoxyribonuclease I
EAM	experimental autoimmune myocarditis
GPX4	glutathione peroxidase 4
GSDMD	gasdermin D
GSDME	gasdermin E
HDL	high density lipoprotein
HF	heart failure
HFmrEF	heart failure with mid-range ejection fraction
HFpEF	heart failure with preserved ejection fraction
HFrEF	heart failure with reduced ejection fraction
HMDM	human monocyte-derived macrophage
HMGB1	high mobility group box 1 protein
IFN-γ	interferon γ
IL-1β	Interleukin-1β
IL-8	interleukin-8
IRE1a	inositol-requiring enzyme-1 alpha
JAK	Janus kinase
LC3	Light-chain 3
LPS	lipopolysaccharides
LVAD	left ventricular assist devices
MAPK	mitogen-activated protein kinase
MC	mast cell
MSC-EVs	mesenchymal stem cell-derived extracellular vesicles
MEK	mitogen-activated extracellular signal-regulated kinase
MI/R	myocardial ischemia–reperfusion
MLKL	mixed lineage kinase domain–like
MPO	myeloperoxidase
mTORC	rapamycin complex
NADPH	nicotinamide adenine dinucleotide phosphate
NE	neutrophil elastase
NET	neutrophil extracellular trap
NF-κB	nuclear factor kappa-B
NK cell	natural killer cell
NLRP	nucleotide-binding oligomerization domain-like receptor pyrin domain-containing
NOX	NADPH oxidase
ox-LDL	oxidized low-density lipoprotein
PAD4	peptidylarginine deiminase 4
PAK2	p21-activated kinase 2
PCI	percutaneous coronary intervention
PD-L1	programmed cell death 1 ligand 1
PI3K	phosphatidylinositol-3-kinase
PKC	protein kinase C
PMA	phorbol myristate acetate
PMN	polymorphic mononuclear neutrophils
RA	rheumatoid arthritis; RBC, red blood cell
RIP3	receptor-interacting protein 3
RIPK	receptor-interacting protein kinase
ROS	reactive oxygen species
SK	small conductance potassium
SLE	systemic lupus erythematosus
STAM	nonalcoholic steatohepatitis induced by neonatal streptozotocin and high-fat diet
STAT3	signal transducer and activator of transcription 3
STEMI	ST-elevation myocardial infarction
STING	stimulator of interferon genes
SYK	spleen tyrosine kinase
TAC	transverse aortic constriction
TLR2	toll-like receptor 2
TLR-9	toll-like receptor 9
TNF	tumor necrosis factor
tPA	tissue plasminogen activator
VTE	venous thromboembolism
